# Emergency management of colon adenocarcinoma in an adolescent female: A rare case report

**DOI:** 10.1002/ccr3.7930

**Published:** 2023-09-18

**Authors:** Chintapenta Harshini, T. Sri Harsha, Billakanti Rajkumar, Humaira Shaikh, Nooreen Habib, Ayush Anand

**Affiliations:** ^1^ Department of Surgery SVS Medical College Yenugonda India; ^2^ Shadan Hospital and Institute of Medical Sciences Hyderabad India; ^3^ B. P. Koirala Institute of Health Sciences Dharan Nepal

**Keywords:** adjuvant chemotherapy, case report, colorectal neoplasms, ileostomy, intestinal obstruction, laparotomy

## Abstract

**Key Clinical Message:**

Colorectal carcinoma (CRC) should be suspected in pediatric patients with a bowel obstruction in an emergency setting. Evidence‐based surgical management with chemotherapy is crucial to prevent adverse outcomes.

**Abstract:**

CRC should be suspected in pediatric patients presenting to the emergency with unspecified abdominal pain. An erect X‐ray abdomen and a colonoscopy should be initial diagnostic tests. If colonoscopy raises suspicion of CRC, a biopsy during colonoscopy is indicated. A computed tomography scan of the chest, abdomen, and pelvis will also aid in diagnosis, staging, and planning intervention. In advanced cases, the intervention includes proximal diversion, bypass, and bowel resection with anastomosis. Sometimes postoperative chemotherapy may be required.

## Graphical abstract

Incisional biopsy of the tumor, peritoneal deposits, and excised specimen.

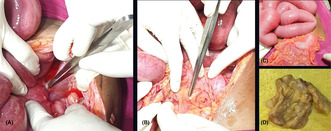

## 1 INTRODUCTION

Colorectal carcinoma (CRC) is the third most common cancer globally, accounting for approximately 1.9 million new cases and 930,000 deaths in 2020.^1^, ^2^ Studies have found an increasing trend of CRC in South Asia.^1^, ^3^, ^4^ However, the data regarding the incidence of CRC in the pediatric population is sparse. The clinical presentation in the pediatric population includes abdominal pain, altered bowel habits, weight loss, and obstruction.^5^, ^6^ In these patients, colonoscopy can be an effective screening tool before going for detailed diagnostic modalities.^7^, ^8^ Early diagnosis and intervention are crucial as delay in diagnosing colon carcinoma may lead to a poor prognosis.^6^ Management of CRC depends upon the American Joint Committee on Cancer (AJCC) staging.^9^ The surgical intervention approach varies in elective and emergency scenarios.^9^, ^10^ Herein, we present the rare case of an adolescent Asian female with colon adenocarcinoma managed with emergency exploratory laparotomy with diversion ileostomy.

## 2 CASE REPORT

A 12‐year‐old Asian female presented with abdominal pain and constipation over the last 3 weeks, with three episodes of vomiting for the past 3 days. There was no history of weight loss or anorexia. There was no history of any chronic illness/comorbidities. Her personal, psychosocial, and family histories were unremarkable. On examination, her vitals were stable, and she had pallor. Her abdominal examination revealed tenderness over the left iliac fossa, hypogastric, and umbilical regions. Per rectal examination revealed stool loaded with increased sphincter tone. The rest of the systemic reviews were unremarkable.

**FIGURE 1:**
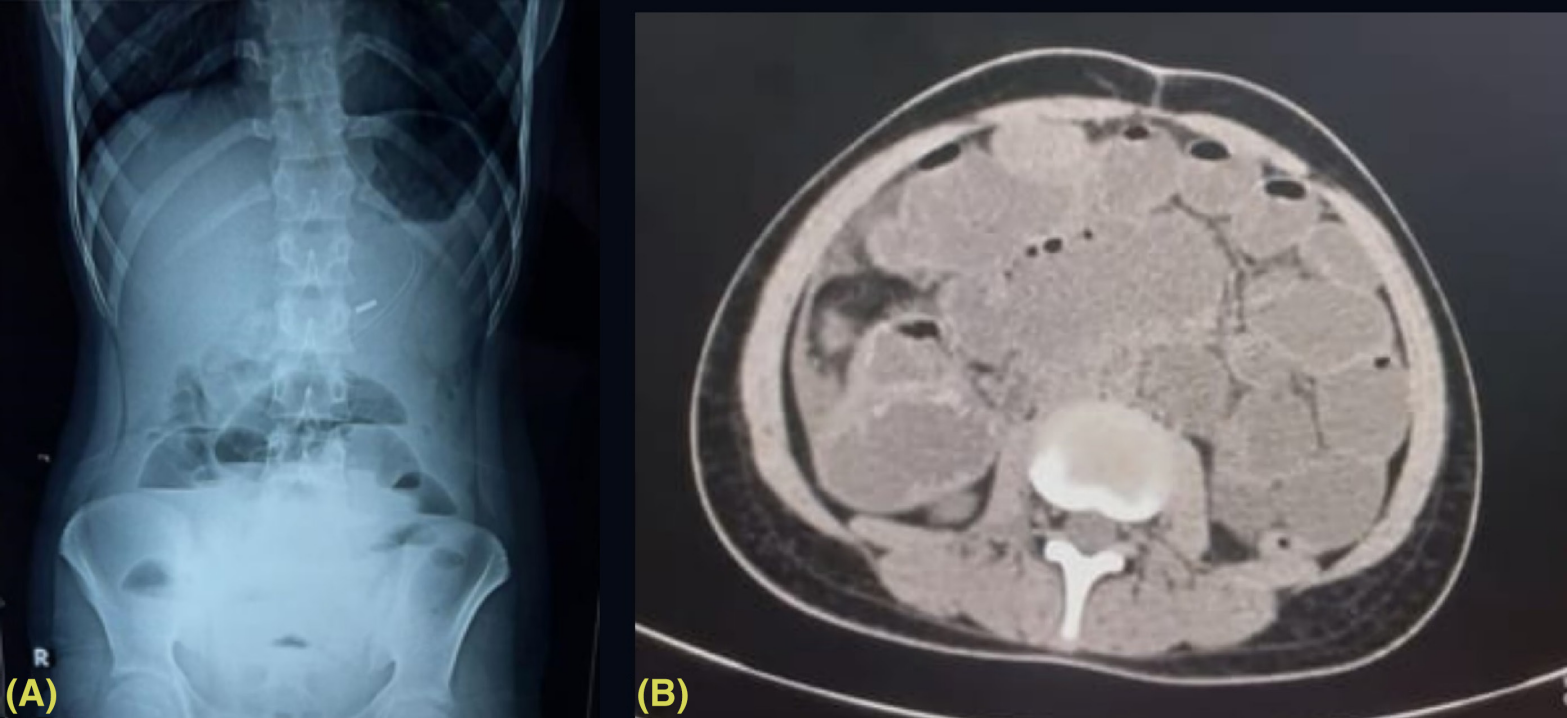
(A) Chest X‐ray of patient. (B) Computed tomography scan of the abdomen showing increased wall thickness near the hepatic flexure.

**FIGURE 2:**
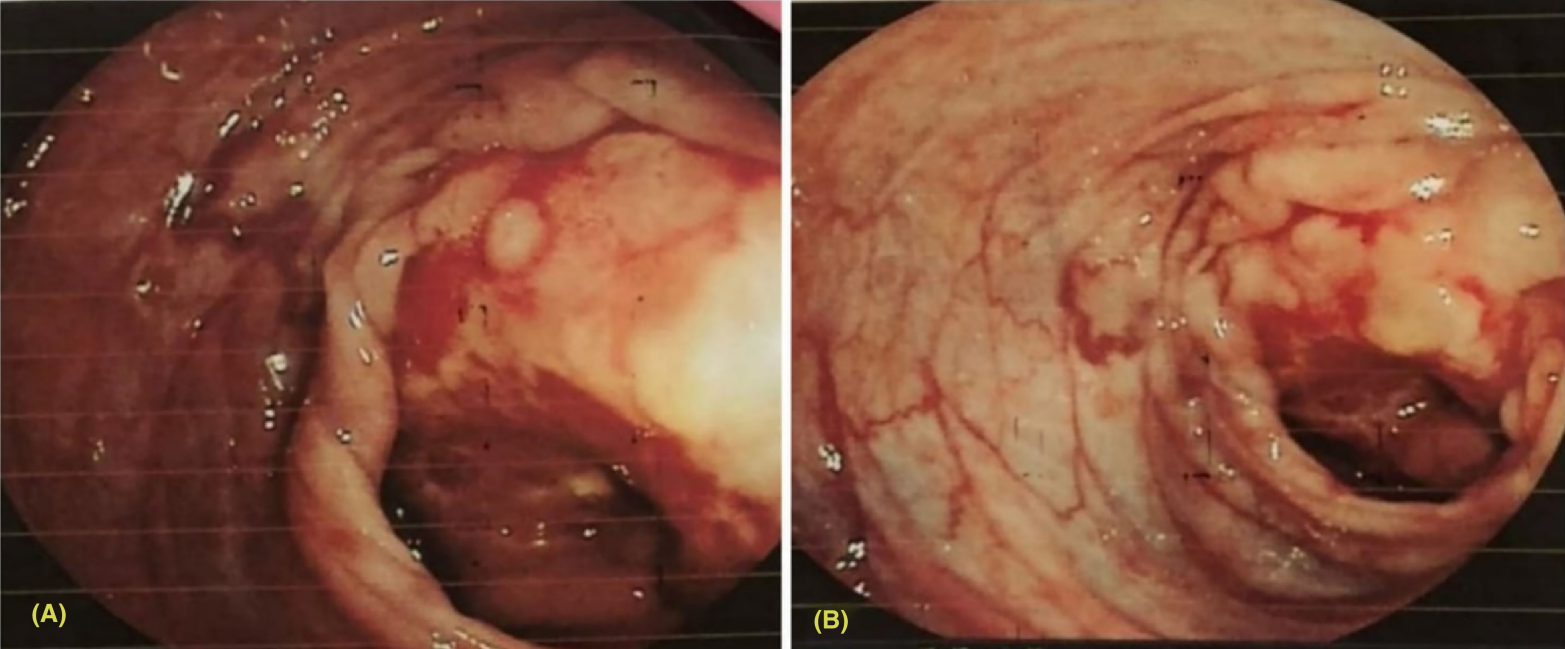
**(**A), (B) Colonoscopy showing mass lesions at the hepatic flexure.

Her laboratory investigations showed anemia, leucocytosis, and stool for occult blood was positive (Table 1). An erect abdominal X‐ray revealed multiple air‐fluid levels (Figure 1A). Following this, we did a colonoscopy, which showed an exophytic mass lesion at the hepatic flexors causing luminal narrowing (Figure 2). The scope could not be passed beyond the lesion. Multiple gray‐white bits measuring 0.5 cm were taken for frozen biopsy from the colon. Also, a computed tomography scan of the abdomen revealed increased wall thickness near the hepatic flexure (Figure 1B).

**TABLE 1:** Laboratory investigations of the patient.

Investigations (unit)	Results	Reference range
Hemoglobin (g/dL)	6.6	11–14
Platelet count (lacs/mm^3^)	5.01	1.5–4.1
Total leucocyte count (μL)	13,800	4000–10,000
Neutrophils (%)	82	40–80
Stool for occult blood	Positive	‐
Carcinoembryogenic antigen (ng/mL)	10.45	0–2.5

**FIGURE 3:**
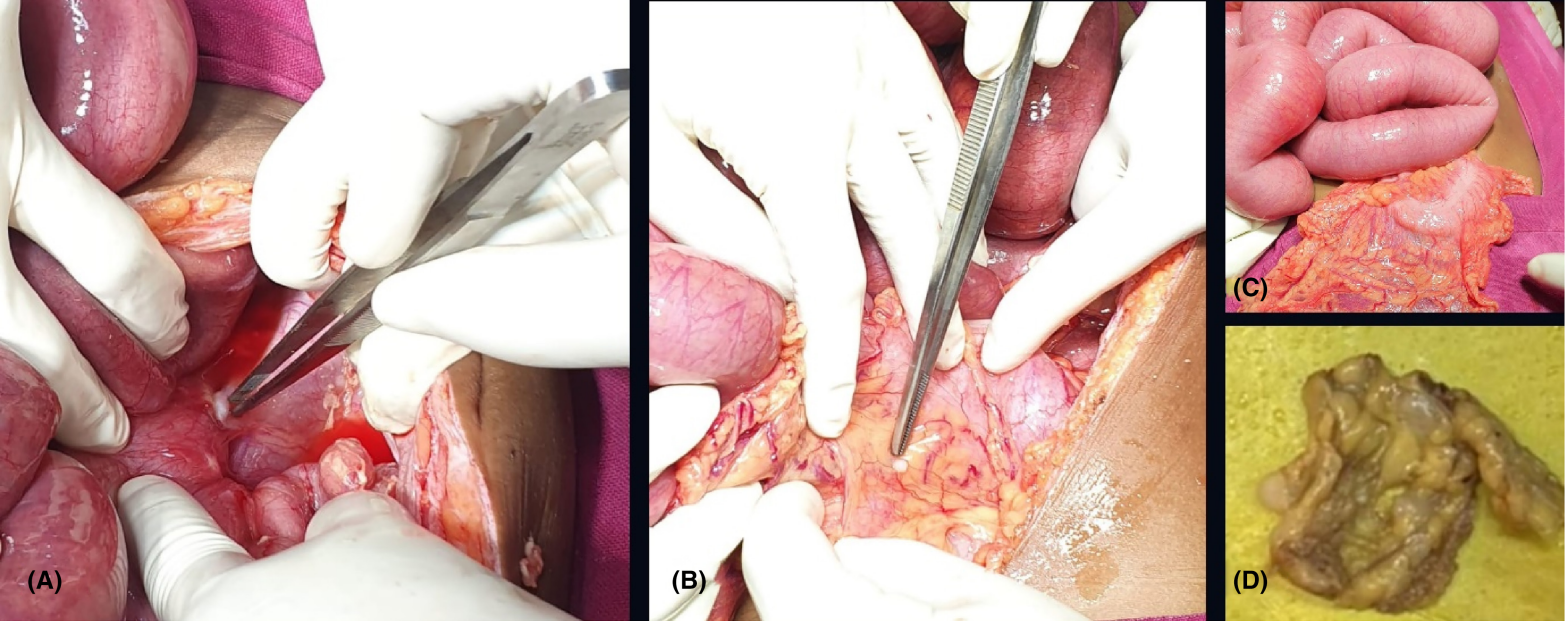
(A) Incisional biopsy of the tumor. (B), (C) Peritoneal deposits. (D) Excised specimen.

To manage acute obstipation, a team consisting of a surgical oncologist, general surgeons, and a medical oncologist decided to go with an emergency exploratory laparotomy, which revealed a relatively mobile growth at the hepatic flexure. Deposits of size 2.5 × 2 cm (Figure 3) were seen in the right peritoneum, pelvic peritoneum, ileum, and omentum, with a peritoneal cancer index (PCI) score of 7 out of 39.^11^ Hence, given deep peritoneal deposits and obstruction, we did a diversion ileostomy. Based on these findings, we diagnosed the patient with colon adenocarcinoma with T4b N2 M1c with AJCC staging IV C.

**FIGURE 4:**
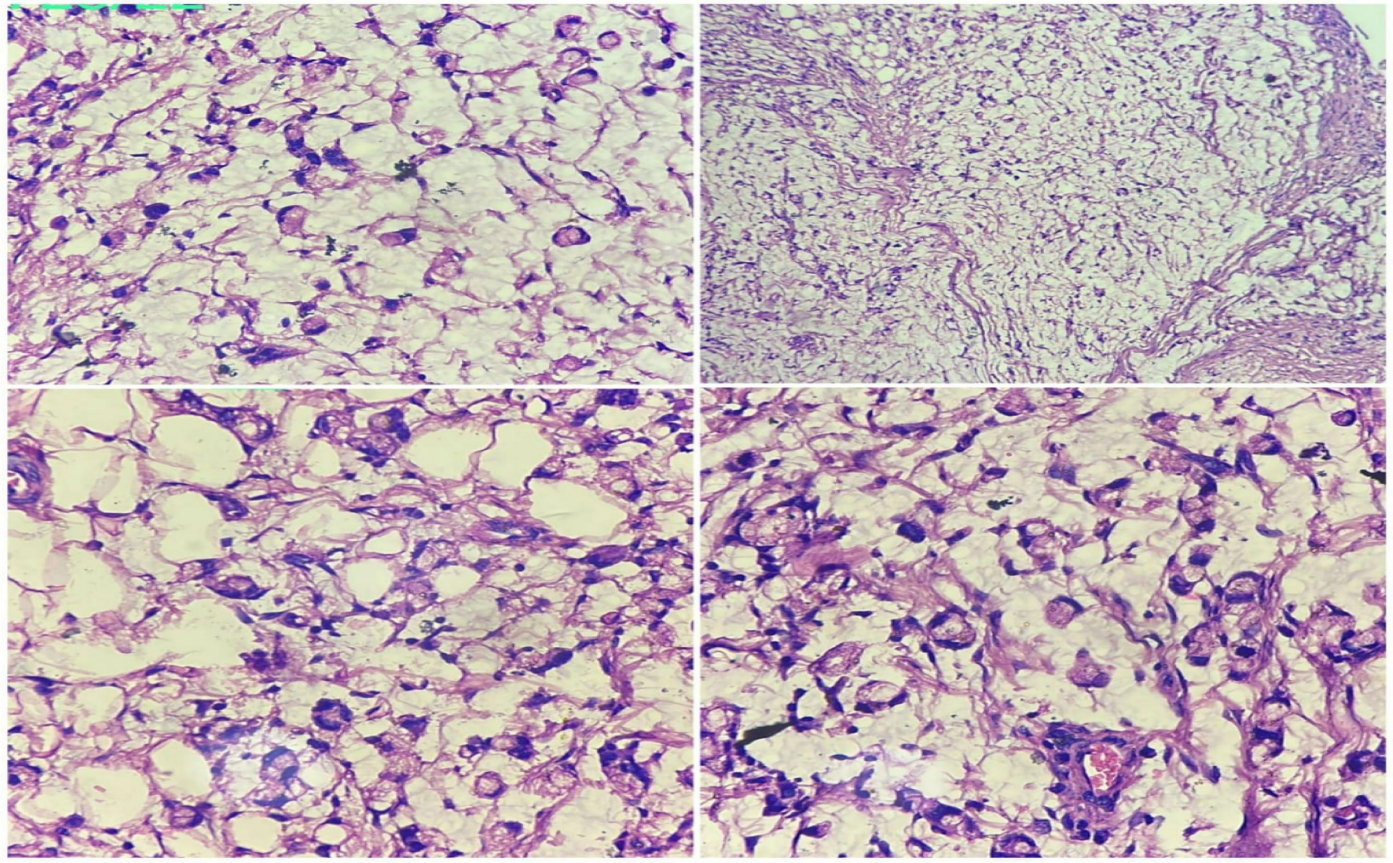
Histopathology of the tumor shows mucinous adenocarcinoma with signet ring cells.

The final histopathology reports (Figure 4) showed normal mucosal glands and tumor tissue in the lamina composed of tumor cells arranged diffusely and in cords. Tumor cells show an altered N:C ratio, abundant eosinophilic cytoplasm, and hyperchromatic and eccentrically placed nucleoli (signet ring cells). Stroma shows acute and chronic inflammatory infiltrate. This suggested colon signet ring type adenocarcinoma with a CEA value of 10.45 ng/mL. It was a grade 4 colon carcinoma. Hence, we started chemotherapy with an injection of oxaliplatin (alkylating agent) 130 mg/m^2^ given intravenously for 21 days, and a tablet capecitabine (antineoplastic) 850 mg/m^2^ per oral was given for 14 days. Each cycle was of 21 days. A total of 6 cycles of chemotherapy were given. The patient was followed up till 6 months post‐intervention, where no significant complications were detected. A follow‐up CT revealed no new developments at the last visit.

## 3 DISCUSSION

In the pediatric age group, CRC is rare and affects approximately one per million population in the USA.^5^ However, the exact incidence of pediatric CRC is unknown in the Asian population. Despite being rare, it is the most common solid primary malignancy of the gastrointestinal tract in the pediatric population.^5^ There are various risk factors that are associated with CRC.^12^ The development of CRC primarily involves adenoma‐carcinoma (70%–90%) and serrated neoplasia pathway (10%–20%).^12^ The diagnosis is made based on presenting signs and symptoms and radiological and histological evaluation.^2^, ^6^ The clinical presentation in the pediatric population is similar to that in adults.^6^ Abdominal pain, vomiting, weight loss, and altered bowel habit are the commonly reported symptoms.^2^, ^5^ Detailed family and past history can further point toward associated risk factors.^12^ In addition, patients can have anemia on laboratory evaluation.^5^, ^12^ Similarly, our patient presented with abdominal pain, altered bowel habit, and vomiting. Also, pallor was detected on physical examination and confirmed as anemia on laboratory investigation. Hence, clinicians in an emergency setting should consider CRC as a differential when a patient has a similar presentation.

Diagnosis can be made based on radiological and histological evaluation.^9^, ^12^ These patients can be initially evaluated with an erect abdomen X‐ray to look for bowel obstruction.^5^, ^10^ Also, a colonoscopy can be used for CRC screening, biopsy, and removal of adenomas before a thorough diagnostic assessment and interventions are planned.^7^, ^8^, ^9^, ^13^ Hence, we initially did an erect X‐ray abdomen, which suggested bowel obstruction. Following this, a colonoscopy showed mass lesions at the hepatic flexure. These findings suggested the possibility of CRC with obstructive symptoms. For further evaluation of CRC, CT chest, abdomen, and pelvis is advised to understand the extent of involvement and decide on the intervention strategy.^2^, ^6^, ^9^, ^12^ We did a CT abdomen and pelvis, which increased wall thickness near the hepatic flexure. After reporting findings during surgical intervention, we made a diagnosis of stage 4 AJCC. Also, biopsy samples were taken during colonoscopy for histological analysis, which later revealed colon adenocarcinoma, the most common histological variant of CRC. Our case showed the use of X‐ray, CT scan and biopsy in accurate diagnosis of CRC.

Poles et al. found that age less than 21 years was significantly associated with mortality in CRC.^14^ Also, CRC in the pediatric population was more aggressive and advanced, with a poor prognosis.^14^ Hence, early diagnosis and intervention are essential for improved outcomes in these patients. Before any surgical intervention, a baseline CEA level should be obtained in CRC.^9^ Hence, we obtained a CEA level before intervention. The intervention approach in CRC varies in elective or emergency settings.^9^, ^10^ In obstruction cases, options include divergent ileostomy and/or colostomy, bowel resection with anastomosis, and bypass.^10^ Any one or combination of these approaches can be used as indicated. Due to obstruction, we opted for an emergency laparotomy with a divergent ileostomy, as the tumor was unresectable. In addition to surgical intervention, postoperative adjuvant chemotherapy may be required in these patients.^14^, ^15^ We also used the CAPOX regimen for our patient. This case showed the evidence based management approach for a patient with unresectable tumor in an emergency setting.

## 4 CONCLUSION

Early diagnosis and intervention in CRC are crucial for better outcomes. CRC should be suspected in pediatric patients with unspecified abdominal pain. The initial evaluation should include an erect X‐ray abdomen and a colonoscopy. If CRC is suspected, a biopsy during colonoscopy should be done. Additionally, a computed tomography scan of the chest, abdomen, and pelvis will help diagnose, stage, and plan intervention. Before planning for surgery, a baseline CEA level should be obtained. Surgical intervention in advanced disease mainly includes proximal diversion, bypass, and bowel resection with anastomosis. Following surgery, postoperative chemotherapy may be required.

## AUTHOR CONTRIBUTIONS

**Chintapenta Harshini:** Conceptualization; data curation; investigation; project administration; writing – original draft; writing – review and editing. **T Sri Harsha:** Conceptualization; data curation; investigation; project administration; writing – original draft; writing – review and editing. **Billakanti Rajkumar:** Conceptualization; data curation; investigation; project administration; supervision; writing – original draft; writing – review and editing. **Humaira Shaikh:** Conceptualization; data curation; investigation; project administration; writing – original draft; writing – review and editing. **Nooreen Habib:** Data curation; investigation; writing – original draft; writing – review and editing. **Ayush Anand:** Conceptualization; investigation; project administration; supervision; validation; visualization; writing – original draft; writing – review and editing.

## Funding information

The authors did not receive any funding for this article.

## CONFLICT OF INTEREST STATEMENT

The authors have no conflict of interest to declare.

## CONSENT

A written informed consent was obtained from the patient's legal guardian based on the journal's policies.

## Data Availability

All relevant data pertaining to this case is made available within the manuscript.
